# Generation of donor organs in chimeric animals via blastocyst complementation

**DOI:** 10.18699/VJ20.690

**Published:** 2020-12

**Authors:** T.I. Babochkina, L.A. Gerlinskaya, M.P. Moshkin

**Affiliations:** Institute of Cytology and Genetics of Siberian Branch of the Russian Academy of Sciences, Novosibirsk, Russia; Institute of Cytology and Genetics of Siberian Branch of the Russian Academy of Sciences, Novosibirsk, Russia; Institute of Cytology and Genetics of Siberian Branch of the Russian Academy of Sciences, Novosibirsk, Russia

**Keywords:** chimerism, interspecies chimera, embryo SC, iPSC, CRISPR/Cas9, organ generation, химеризм, межвидовой химеризм, ЭС клетки, ИПСК, CRISPR/Cas9, органы для трансплантаций

## Abstract

The lack of organs for transplantation is an important problem in medicine today. The growth of organs in chimeric animals may be the solution of this. The proposed technology is the interspecific blastocyst complementation method in combination with genomic editing for obtaining “free niches” and pluripotent stem cell production methods. The CRISPR/Cas9 method allows the so-called “free niches” to be obtained for blastocyst complementation. The technologies of producing induced pluripotent stem cells give us the opportunity to obtain human donor cells capable of populating a “free niche”. Taken together, these technologies allow interspecific blastocyst complementation between humans and other animals, which makes it possible in the future to grow human organs for transplantations inside chimeric animals. However, in practice, in order to achieve successful interspecific blastocyst complementation, it is necessary to solve a number of problems: to improve methods for producing “chimeric competent” cells, to overcome specific interspecific barriers, to select compatible cell developmental stages for injection and the corresponding developmental stage of the host embryo, to prevent apoptosis of donor cells and to achieve effective proliferation of the human donor cells in the host animal. Also, it is very important to analyze the ethical aspects related to developing technologies of chimeric organisms with the participation of human cells. Today, many researchers are trying to solve these problems and also to establish new approaches in the creation of interspecific chimeric organisms in order to grow human organs for transplantation. In the present review we described the historical stages of the development of the blastocyst complementation method, examined in detail the technologies that underlie modern blastocyst complementation, and analyzed current progress that gives us the possibility to grow human organs in chimeric animals. We also considered the barriers and issues preventing the successful implementation of interspecific blastocyst complementation in practice, and discussed the further development of this method

## Abstract (ru)

Сегодня актуальной проблемой в медицине является нехватка органов для трансплантаций. Одна из предполагаемых технологий получения этих органов – выращивание их из клеток человека в организме химерных животных с использованием метода межвидовой бластоцистной комплементации в комбинации с методами геномного редактирования и получения плюрипотентных стволовых клеток. Метод CRISPR/Cas9 позволяет создавать животных для бластоцистной комплементации с так называемыми свободными нишами. Совершенствование методов получения индуцированных плюрипотентных стволовых клеток дает возможность получать донорские клетки человека, способные заселять свободную нишу. Таким образом, с помощью современных технологий можно осуществить межвидовую бластоцистную комплементацию между человеком и другими животными, что в будущем позволит выращивать органы человека внутри химерных животных. Однако на практике для проведения успешной межвидовой бластоцистной комплементации необходимо решить ряд проблем: усовершенствовать методы получения «химер-компетентных клеток», преодолеть специфические межвидовые барьеры, подобрать совместимые стадии развития клеток для инъекции и соответствующего этапа развития эмбриона-реципиента, предотвратить апоптоз донорских клеток, добиться эффективной колонизации донорскими клетками человека организма животного-реципиента. Кроме того, очень важно проанализировать и законодательно урегулировать этические аспекты, возникающие при разработке технологий, связанных с получением химерных организмов с участием клеток человека. Многочисленные исследования направлены на решение этих проблем, а также на поиски новых подходов в создании межвидовых химерных организмов с целью выращивания органов человека для трансплантаций. В настоящем обзоре описаны исторические этапы развития технологии бластоцистной комплементации, детально разобраны методы, лежащие в основе ее современного варианта, и проанализированы достижения, позволяющие приблизиться к возможности выращивания органов человека в химерных животных. Рассмотрены также барьеры и проблемы, мешающие успешному применению данного подхода на практике, и дальнейшие перспективы его развития.

## Chimerism: definitions and classifications

The first studies on generating chimeric animals were carried out in the 60s of the last century (Tarkowski, 1961; Mintz, 1965; McLaren, Bowman, 1969). Since then, a significant amount of scientific knowledge on chimerism has been accumulated, modern definitions have been formulated and various classifications of chimeras have been proposed.

Chimeric animals are composed of genetically different cells originating from two or more different zygotes (Tippett, 1983). There are different classifications of chimerism, depending on the number and type of donor cells and their distribution in chimeric organisms. Chimerism can be natural or artificial. Natural chimerism is represented by two forms: tetragametism and microchimerism. Tetragametism results from the fertilization of two separate eggs by two different spermatozoa, followed by the development of a single organism with mixed cell lines (Drexler et al., 2005). Microchimerism is a phenomenon that occurs when a small number of cells from another individual are present in a multicellular organism. Examples of natural microchimerism are twin chimerism (Chen K. et al., 2013) and feto-maternal microchimerism (Nelson et al., 1998). The artificial chimerism occurs for example as a result of organ or tissue transplantation or blood transfusions. 

The chimerism can be partial or systemic depending on the degree of donor cells distribution in a chimeric organism (Suchy, Nakauchi, 2017). For example, during organ or tissue transplantation the distribution of donor cells is limited to a particular organ or tissue which results in partial chimerism. Systemic chimerism can be observed, for example, during the fusion of embryos at an early stage of development. As a result of such a fusion, an embryo with cell lines which distributed over different organs and tissues is formed, with these lines originating from two different zygotes

Chimerism can be primary and secondary. Primary chimerism occurs in the early stages of embryogenesis, and secondary chimerism occurs after the onset of gastrulation (Mascetti, Pedersen, 2016a, b). Chimerism can be intraspecies and interspecies. The intraspecies chimeras consist of cell lines originating from different zygotes of the same species. Interspecies chimeras consist of cell lines originating from two or more zygotes of representatives of different species.

## The methods underlying the development of the blastocyst complementation 

The most popular methods to obtain chimera under laboratory conditions are cell aggregation (Tarkowski, 1961) and microinjection into the embryo (Gardner, 1968). Aggregation methods for producing chimeras are technically easier, do not require expensive micromanipulation equipment, and sometimes can work more efficiently than injection methods (Tachibana et al., 2012). However, in some cases, for example, when obtaining interspecies chimeras, the trophectoderm with donor cells can impede implantation, and in this case injection methods are preferred (MacLaren et al., 1992). In addition, the injection methods allow to control the number of injected cells.

In their study Okumura and colleagues compared the degree of distribution of rat cells in chimeric rat-mouse embryos by different methods: the 8-cell aggregation method, injection into an 8-cell embryo, and injection into a blastocyst. According to the study, the degree of chimerism was highest when researchers used the injection method into an 8-cell embryo, although the percentage of chimeric mice was higher when they injected cells into the blastocyst (Okumura et al., 2019).

The most common and promising method to generate human organs for transplantation in the organisms of interspecies chimeric animals is injection into the blastocyst – so called the blastocyst complementation method. Further in this review this method is considered first in its application to the rodents, then the development of techniques related to this method is described: obtaining “free niches” of animals and obtaining “chimera-competent” human cells. These techniques made it possible to perform the interspecies blastocyst complementation between humans and other animals.

## The early version of the blastocyst complementation for obtaining rodent chimeras

Intraspecies chimeras. In 1993 the method of intraspecies blastocyst complementation was successfully demonstrated for the first time. The main idea of the method was that wild type mouse embryonic stem (ES) cells were injected into the blastocyst derived from Rag2 –/– immunodeficient mouse with T and B lymphocytes deficiency. As a result, donor T and B lymphocytes were observed in chimeric animals (Chen J. et al., 1993). An important result of this study was that donor ES cells were able to differentiate into T and B lymphocytes, using the vacant lymphoid T and B cell niche in an immunodeficient organism. It demonstrated the possibility of generating organs in the body of chimeric animals with so-called “free niches”. Then, in 2007, blastocyst complementation was used to grow pancreatic epithelium in Pdx1 –/– deficient mice with impaired pancreas development (Stanger et al., 2007). In 2012, successful intraspecies blastocyst complementation of ES cells from a healthy mouse into the blastocyst of a Sall1 –/– deficient mouse with impaired renal development was demonstrated (Usui et al., 2012).

Interspecies chimeras. In 2010, for the first time viable interspecies chimeras with a developed rat pancreatic epithelium were obtained in the body of a Pdx1 –/– deficient mouse by blastocyst complementation (Kobayashi et al., 2010). In this study, scientists successfully injected rat pluripotent ES cells into murine Pdx1 –/– blastocysts that were genetically modified to impair pancreas development. In 2011, interspecies blastocyst complementation was used to inject rat ES cells into the blastocyst of a nude mouse without a thymus, and a chimeric mouse with a functioning thymus of rat origin was obtained (Isotani et al., 2011). Recently, it was reported about the successful generation of a mouse kidney in the chimeric organism of Sall1 –/– rat by interspecies blastocyst complementation (Goto et al., 2019).

In 2017, a Nakauchi group demonstrated the successful transplantation of pancreatic tissue generated from pluripotent stem cells in Pdx1 –/– deficient rats to diabetic mice (Yamaguchi et al., 2017). These results proved the possibility of using tissues generated in the body of interspecies chimeric animals for organ transplantation.

Further in the review, the following technologies underlying modern blastocyst complementation are discussed in details: obtaining animals with so-called “free niches” and obtaining “chimera-competent” cells for injection into the blastocyst.

## Generation of animals with “free niches”

The animals with “free niches” in organogenesis, that is with the absence or partial development of certain organs or special cell lines, are necessary for obtaining chimeric animals by the method of blastocyst complementation. Such animals with “free niches” in organogenesis are possible to obtain by turning off the expression of genes involved in organogenesis. Certain types of stem cells in these animals lose the ability to specialize, proliferate or differentiate, that is, they cannot participate in organogenesis and the organ does not develop

When donor cells with normal organogenesis are injected into the blastocyst of animals with “free niches”, missing organs can be formed. For the generation of donor organs in chimeric organisms, it is necessary that the donor’s cells have an advantage in the organogenesis of a certain tissue or organ, since these cells are introduced in small numbers, and they do not initially have a selective advantage. The creation of “free niches” allows donor cells to proliferate without competition with host cells in a chimeric organism and to form a given organ. A “free niche” can be created by the gene knockout method (Offield et al., 1996; Ohinata et al., 2005) or by methods of genome editing: zinc finger nucleases(ZFN), TALE-associated nucleases (transcription activator-like effector nucleases, TALEN) and CRISPR/Cas9 (clustered, regularly interspaced, short palindromic repeats (CRISPR)/CRISPR-associated system (Cas))

The obtaining of knockout mice by injecting messenger RNA (mRNA) nuclease into mouse zygotes (based on the ZFN method) was demonstrated in 2010 (Carbery et al., 2010). The knockout mice were obtained in 2013 using the TALEN technology by injecting TALEN mRNA into the cytoplasm of zygotes (Sung et al., 2013). The CRISPR/ Cas9 method was also first demonstrated in 2013 (Cong et al., 2013; Mali et al., 2013); this method today is the most popular in genetic engineering. In this method, targeted genome editing is carried out due to the complementary interaction between the non-coding synthetic RNA and the DNA of the target sites. This forms a complex of non-coding RNAs and Cas proteins which have nuclease activity. The pigs with a mutation in the genes were obtained by using the CRISPR/Cas9 method in the cells of pig embryo at the blastocyst stage in vitro in 2014 (Whitworth et al., 2014). Successful intraspecific neural blastocyst complementation was performed for the first time in 2018 in mice with “free niches” in the brain, including those obtained using the CRISPR/Cas9 method (Chang et al., 2018).

## Types of “chimera-competent” cells for injection into a blastocyst

In order to obtain chimeric animals, ES cells and induced pluripotent stem cells (iPSCs) are used.

ES cells. Pluripotent cells isolated from the inner cell mass(ICM) and epiblast of the embryo are the most suitable candidates to generate organs in chimeric animals, since they are able to differentiate into the embryonic tissue. It turned out that the pluripotency degree of pluripotent cells of ICM and epiblast are different in mice. It was proposed to call “true” pluripotent cells obtained from ICM at an early pluripotency stage “naïve”, and epiblast cells obtained at a later pluripotency stage – “primed” (Nichols, Smith, 2009; Hanna et al., 2010). In addition, it turned out that pluripotent cells isolated at the same developmental stage in different species differ in the degree of pluripotency. For example, mouse ES cells isolated from the ICM relate to the “naïve” status, while similar human cells relate to the “primed” status of pluripotency.

Pluripotent ES cells in the “naïve” status, isolated from the ICM of the blastocyst before the implantation stage, are of the most interest for obtaining chimeric animals since it turned out that cells in the “primed” status are not able to take part in the formation of chimeras when they are injected into the preimplantation blastocyst (Tesar et al., 2007).

Mouse ES cells were first obtained in 1981 (Evans, Kaufman, 1981). Mouse ES cells exhibit typical characteristics of pluripotency: they have the ability to form cells of ecto dermal, mesodermal, and endodermal origin (Martin, 1981), and they are involved in the formation of all tissues of the adult organism, when injected into the blastocyst (Bradley et al., 1984; Hayashi et al., 2017). And most importantly, mouse ES cells are involved in the formation of chimeras after injection into the blastocyst (Nichols, Smith, 2009; Betschinger et al., 2013).

In 1995, ES cells of Rhesus macaque were first obtained (Thomson et al., 1995). The same researchers obtained human ES cell lines from preimplantation human embryos for the first time in 1998. The in vivo pluripotency test demonstrated the ability of human ES cells to form teratomas with tissues of endodermal, mesodermal, and exodermal origin (Thomson et al., 1998). The differentiation of ES cells with the formation of embryoid bodies and differentiation into various cell types was shown in vitro for human ES cells (Wobus, Boheler, 2005). It is impossible to test for chimerism and to perform an accurate assessment of the pluripotency of human and primate ES cells due to ethical reasons. It appeared that although the human ES cells are similar in a number of characteristics to mouse ES cells (Wobus, Boheler, 2005; Huang et al., 2014), they differ significantly from them (Friel et al., 2005; Watanabe et al., 2007). It is assumed that human ES cells belong to the “primed”, and mouse ES cells belong to the “naïve” status of pluripotency

Pluripotent ES cells of humans and primates in “naïve” status. Human ES cells in “naïve” status were first obtained in 2010 by the method of ectopic induction of the factors Oct4, Klf4, and Klf 2 in combination with LIF and the inhibitors GSK3β and ERK1/2 (Hanna et al., 2010). Then, the cultivation medium and cultivation conditions were optimized (Gafni et al., 2013). Attempts have also been made to obtain pluripotent cells in the “naïve” status in primates (Fang et al., 2014; Chen Y. et al., 2015; De Los Angeles et al., 2019). Today, numerous studies are aimed at obtaining pluripotent ES cells in “naïve” status and maintaining this status under culture conditions (Liu et al., 2017; Kilens et al., 2018). Due to ethical reasons, it is not possible to test the “naïve” status of these human pluripotent cells, but it is possible to determine the putative criteria by which these cells could be considered pluripotent in “naïve” status. Today, the so-called “naïve” factors of pluripotency are already described. One of these factors is KLF4, which is specific for mouse “naïve” pluripotent stem cells and for human preimplantation embryos (Guo et al., 2009; Dunn et al., 2014; Boroviak et al., 2016). In addition, cells in the “naïve” status are characterized by nuclear localization of TFE3 and a high level of mitochondrial respiration (Zhou et al., 2012; Betschinger et al., 2013). Other researchers have demonstrated that the level of transcription of transposons corresponds to the status of pluripotency; in addition, the induction of the “naive” cell status is accompanied by DNA hypomethylation (Theunissen et al., 2016; Wang, Li, 2017)

Obtaining “naïve” status in somatic cells. iPSC. Simultaneously with the study of the “naïve” status of pluripotent ES cells, the technologies for the production of iPSCs from somatic cells were actively developing. The iPSCs are a new type of pluripotent cells that can be obtained by reprogramming differentiated somatic cells. For the first time iPSCs from somatic cells were obtained by exogenous expression of transcription factors in 2006 (Takahashi, Yamanaka, 2006). The essence of the method is the transfection of an adult cell with four genes (Oct4, Sox2, Klf4 and c-Myc), which encode transcription factors associated with the pluripotent status of embryonic cells. Researchers were able to obtain human iPSC cell lines that meet all the criteria for ES cells from human skin fibroblasts (Takahashi et al., 2007; Yu et al., 2007) and from human skin keratocytes (Aasen et al., 2008). Since ectopic expression of the c-Myc and Klf4 genes is undesirable due to the high risk of forming malignant tumors, these genes were successfully replaced with the less dangerous genes Nanog and Lin28 in 2007 (Okita et al., 2007; Yu et al., 2007).

The iPSC cells are very similar to ES cells: similar morphology and growth profile, and the same culture conditions (growth factors and signaling molecules). The iPSCs retain the normal karyotype during cultivation, have high telomerase activity, and differentiate in vitro into tissue cells of all three germ layers (Yu et al., 2007).

Capabilities and limitations of using “naïve” ES cells and iPSCs. The unique properties of ES cells and iPSCs make it possible to obtain “chimera-competent” cells for blastocyst complementation. When ES and iPSCs are injected into the blastocyst, these cells are included into development, leading to the formation of animals with a high degree of chimerism. The properties of ES cells and human iPSCs make them an exceptional source for obtaining tissues and organs in transplantation and create prospects for the development of new approaches for the treatment of incurable diseases. The technology for generation iPSCs also demonstrates the possibilities for generation autologous stem cells, which in the future will allow to solve the problem of immunological compatibility during transplantation of organs from chimeric animals to a patient. In addition, this technology makes it possible to obtain pluripotent stem cells from various types of somatic cells, thus avoiding the ethical issues associated with the use of living embryos.

However, there are some limitations. The cultured ES cells and iPSCs vary significantly in their pluripotent differentiation potential and gene expression profile (Yu et al., 2007). In the population of the obtained ES cells and iPSCs, undifferentiated cells remain which can give rise to a tumor or reactivation of viruses. It also remains a problem to obtain a large number of “chimera-competent” cells of high quality suitable for clinical use. In addition, heritable epigenetic disorders were found in cultured ES cells, which may be associated with the development of hereditary diseases and carcinogenesis (Allegrucci et al., 2007). Consequently, there is a necessity to standardize the condition for obtaining, cultivating, and assessing the pluripotent status of iPSCs and ES cells. 

## Application of the modern method of blastocyst complementation

Interspecies chimeras of humans and rodents. The availability of “chimera-competent” human cells, generating the animals with “free niches” in organogenesis and obtaining interspecies chimeras of animals by the method of blastocyst complementation made it possible to make attempts to create chimeric organisms between humans and other animals. In 2006 for the first time, human ES cells at the early stages of embryogenesis were injected into a mouse blastocyst; the obtained chimeras showed developmental abnormalities (James et al., 2006). In 2013, chimeric mice were obtained by injecting human iPSCs; however, for ethical reasons, the mouse embryos were sacrificed at an early stage of development (Gafni et al., 2013). Then, in 2014, chimeric animals were obtained by microinjection of “naïve” iPSCs obtained from Rhesus macaque fibroblasts into a mouse embryo at the blastocyst stage (Fang et al., 2014).

However, in the obtained interspecies chimeras, the degree of revealed chimerism was low, especially in comparison with the degree of chimerism in intraspecies chimeras among rodents. It is speculated that this might be due to the evolutionary distance between humans and other animals. Interestingly, attempts to obtain an interspecies human chimera were successful when human iPSCs were injected into a mouse embryo at a later stage of embryonic development – at the gastrula stage (Mascetti, Pedersen, 2016b). Thus, the ability to form chimeras depends on the coordination of the in vitro developmental stages of donor cells with the in vivo embryo developmental stages.

Interspecies chimeras of humans and large domestic animals. In 2017, chimeric embryos were obtained between a human and a pig, as well as between a human and a cow (Wu et al., 2017). In this study, the researchers used CRISPR/ Cas9 genetic editing to create a “free niche” in combination with blastocyst complementation. Their results demonstrated that “naïve” human pluripotent stem cells proliferate in porcine and bovine preimplantation blastocysts, while their ability to proliferate is limited in porcine postimplantation blastocysts. Interestingly, with the use of so-called “intermediate human pluripotent stem cells”, the degree of chimerism and the ability to proliferate into various cell types in post-implantation pig embryos was higher (Tsukiyama, Ohinata, 2014; Wu et al., 2017). Recently, the creation of a chimeric embryo between Macaca fascicularis and a pig was reported, functioning donor ES cells of the primate were detected in the tissues of the pig (Fu et al., 2020).

## Artificial embryo

The creation of an artificial embryo is a promising alternative to the use of animal and human embryos for research purposes. Different researchers have demonstrated the creation of embryo-like formations on stem cell culture (Pera et al., 2015; Harrison et al., 2017). In 2017, the possibility of creating artificial embryos was demonstrated by the aggregation of trophoblastic stem cells and totipotent ES cells, which independently assemble into a blastocyst on a substrate of a three-dimensional extracellular matrix. Scientists have shown that the development of the embryo, its morphogenesis, structure and cellular composition follow the same development patterns as in a normal embryo (Harrison et al., 2017)

Then, in 2018, a fully-fledged blastocyst model was created, which was called the blastoid (Rivron et al., 2018). Recently, three main types of stem cells have been obtained from fibroblasts: epiblast cells, primitive endoderm cells, and trophectoderm cells. To obtain a certain type of these pluripotent cells, a combination of five transcription factors was selected: Gata3, Eomes, Tfap2c, Myc, and Esrrb. This achievement could lead to the creation in vitro of fully-fledged artificial embryos without the use of an egg and a sperm cell (Benchetrit et al., 2019). Advances in the creation of an artificial embryo demonstrate the possibility of using it to obtain chimeric organisms in the future.

## The main problems hindering the development of technologies for generating organs in chimeric animals, and possible ways to solve them

Growing rat organs in a mouse organism and generation of man-pig, man-cow chimeras give us the possibility of creating xenogeneic organisms among various animal species and generating human organs in the future. The candidate animals for organ transplant growing considered are pigs, cows, sheep, and primates.

The development of technology for farming human organs in xenogeneic animals such as pigs is hindered by a number of factors. There is a risk of zoonosis and the risk of contamination of human organs with cells or proteins of the recipient animal (Rashid et al., 2014; Matsunari et al., 2020). One problem is that retroviruses integrated into the genome of chimeric animals can be transferred to humans when growing human organs. The consequences of the incorporation of animal retroviruses into the human genome cannot be predicted. There are fears that human organs derived from chimeric animals could be a source of danger.

In addition, there are a number of poorly identified and poorly understood biological factors associated with differences in the rate of embryonic development in different species (Barry et al., 2017). Understanding the mechanisms of these differences, the ability to modulate the time and developmental stage of donor cells in vitro, and the ability to influence the developmental stage in vivo would allow the synchronization of donor and host cells in a chimeric model. Recent studies have shown that the synchronization of developmental stages between donor cultured pluripotent ES cells and the recipient is a significant criterion for the successful formation of a chimera. For example, “naïve” mouse ES cells are involved in the formation of a chimera only when injected at the blastocyst stage, while “primed” mouse ES cells isolated from the epiblast are involved in the formation of a chimera when injected at the gastrula stage (Huang et al., 2012).

It is also interesting that attempts to obtain an interspecies human chimera were successful when the injection was carried out at a later stage of embryonic development. Successful microinjection of human iPSCs into a mouse embryo at the gastrula stage was demonstrated in 2016, which confirms the hypothesis that the ability to form chimeras depends on the coordination of the in vitro stages of donor cells with the stage of in vivo host embryo development (Mascetti, Pedersen, 2016b).

One of the problems of generating interspecies human chimeras is the low percentage of donor cells in the chimeric organism. It is assumed that the negative results and low degree of chimerism in experiments on generating chimeras are associated with the apoptosis of cells. In 2016, it was demonstrated that expression of the anti-apoptotic gene Bcl2 in “chimera-incompetent” epiblast stem cells in rat allows these cells to turn into “chimera-competent” cells and participate in the formation of all tissues in a chimeric rat-mouse embryo when injected into a mouse blastocyst (Masaki et al., 2016).

Very recently, it became possible to create human-mouse chimeric embryos in which the proportion of human cells for the first time was 4 %. In this study, «naïve» human PSCs obtained by the inhibition of mTOR protein kinase were microinjected into mouse blastocysts (Hu et al., 2020).

Another important problem to be solved for the successful cultivation of donor human organs in chimeric organisms is the problem of organ vascularization. Previous studies have demonstrated that vessels in chimeric organisms are formed from the cells of both donor and recipient (Kobayashi et al., 2010; Usui et al., 2012; Yamaguchi et al., 2017). For the successful transplantation of human organs grown in animals, it is necessary for the organ’s circulatory system, like the organ, to be formed from human cells in order to minimize the xenogenic component during transplantation. Many researchers are working on this problem (Hamanaka et al., 2018; Matsunari et al., 2020). To solve all these problems, the factors influencing the success of the colonization of pluripotent donor cells into the organism of the recipient animal, and the mechanisms underlying the differentiation of these cells in the conditions of the “free niche” are still to be determined and investigated.

Besides biological, there are also ethical barriers. For example, one of the issues that can arise with interspecies human-animal chimeras is the production of gametes with the human genome in chimeric animals (Bourret et al., 2016; Farahany et al., 2018). Concerns are also raised by the likelihood of humanization of chimeric animals upon accidental differentiation of human cells in the brain tissues of the recipient (Shaw et al., 2015). In 2019, it was demonstrated that these issues can be solved by disabling the Prdm14 and Otx2 genes responsible for the formation of gametes and the brain in microinjected “chimera-competent” cells (Hashimoto et al., 2019).

## Conclusion

Thus, in order to carry out successful blastocyst complementation and obtain an interspecies chimera between a human and another animal for the purpose of growing organs for transplantation, two key technologies need to be improved: (1) creation of animals with “free niches”, and (2) ethical generation of pluripotent “chimera-competent” human cells capable of differentiating into a target organ or tissue in the body of a host animal. In addition, it is necessary to understand and overcome the biological barriers that cause the absence or low percentage of chimerism of pluripotent “chimera-competent” ES cells in the animal organism. It is also important to regulate emerging ethical issues at the legislative level. Despite all the difficulties, the technology of growing donor organs in chimeric organisms is very promising.

## Conflict of interest

The authors declare no conflict of interest.
